# Tibial plateau fractures are associated with ligamentous and meniscal injuries. Preoperative evaluation of magnetic resonance imaging influences surgical treatment

**DOI:** 10.1007/s00068-024-02581-0

**Published:** 2024-06-26

**Authors:** Salvatore Risitano, Fortunato Giustra, Francesco Bosco, Antonio Rea, Giorgio Cacciola, Raffaella Rizzolo, Luigi Sabatini, Marcello Capella, Alessandro Massè

**Affiliations:** 1 Department of Orthopaedic Surgery and Traumatology, “Città Della Salute E Della Scienza” - CTO Hospital of Turin, 10126 Turin, Italy; 2 Department of Orthopaedics and Traumatology, Ospedale San Giovanni Bosco – ASL Città di Torino, Piazza del Donatore di Sangue, 3, 10154 Turin, Italy; 3 Department of Precision Medicine in Medical, Surgical and Critical Care (Me.Pre.C.C.), University of Palermo, 90133 Palermo, Italy; 4 Department of Orthopaedics and Traumatology, G.F. Ingrassia Hospital Unit, ASP 6, 90131 Palermo, Italy; 5 Department of Orthopaedics, Traumatology and Rehabilitation, University of Turin, CTO, Via Zuretti 29, 10126 Turin, Italy; 6 Department of Orthopaedics and Traumatology, Humanitas Gradenigo Hospital, Turin, Italy

**Keywords:** Schatzker, MRI, Tibial plateau fracture, Knee, Ligament, Meniscus

## Abstract

**Background:**

Tibial plateau fractures (TPFs) are usually associated with ligamentous or meniscal injuries that could remain misdiagnosed. An appropriate and early recognition may change the surgical management of these soft tissue injuries (STIs) that could be addressed concomitantly with TPF treatment. Magnetic resonance imaging (MRI) is an efficient diagnostic test to identify all associated STIs in TPFs. This study aims to analyze the MRI impact in identifying and guiding the STIs treatment in TPFs.

**Material/methods:**

This retrospective study included a consecutive series of 57 patients with TPFs treated between January 1st, 2022, and December 31st, 2022. All fracture patterns were classified according to the AO/OTA and Schatzker classification. The prevalence of STIs, including medial meniscus (MM), lateral meniscus (LM), anterior cruciate ligament (ACL), posterior cruciate ligament (PCL), medial collateral ligament (MCL), and lateral collateral ligament (LCL) injuries, was assessed through the MRI evaluation.

**Results:**

A statistical significance was found regarding the MRI detection of LM, ACL, PCL and MCL injuries that led to additional surgical procedures at the same time as the TPFs treatment (*p* < 0.05). In contrast, the amount of additional MM and LCL injuries identified by MRI, which resulted in other surgical procedures, was not statistically significant (*p* > 0.05).

**Conclusions:**

Preoperative MRI has been demonstrated to be an effective procedure for diagnosing STIs in TPFs, significantly influencing and changing the surgical treatment.

Level of evidence: IV.

## Introduction

Tibial plateau fractures (TPFs) are complex, comminuted intra-articular injuries that are relatively rare and account for 1.0% of all fractures [1]. In young patients, they occur from high-energy trauma, while in elderly one from low-energy injury and are usually associated with ligamentous and meniscal lesions [2]. Soft tissue injuries (STIs) rates, reported in several studies using magnetic resonance imaging (MRI) on patients with TPFs, range from 47 to 99% [3–5]; in a recent systematic review of the literature, at least a ligamentous or meniscal injury was observed in 93% of TPFs [6]. Diagnosing and treating STIs is relevant because suboptimal management may result in poor knee function and persistent instability, requiring reoperation [1, 2, 7–9]. A standard radiographic evaluation, including anteroposterior (AP) and lateral views on radiographs, is usually performed in all patients suspected of a TPF. Afterward, computed tomography (CT) is generally done to analyze the fracture patterns [10]. The currently most widely used TPF classification systems, Schatzker [10], Kfuri [11–13], and Osteosynthesefragen/Orthopaedic Trauma Association (AO/OTA) [13, 14], do not include associated STIs but are based exclusively on fracture analysis [10–14]. However, CT does not provide sufficient preoperative information on associated ligamentous or meniscal injuries, and clinical examination under acute conditions is often limited by patient swelling and pain [12–15]. In recent decades, an increasing MRI application was performed in the acute phase to characterize further STIs, comminution, and occult fracture lines to optimize surgical treatment [16].

This study aims to investigate whether the routinary MRI results in additional surgical procedures to manage STIs that would otherwise remain misdiagnosed and untreated, helping the surgeon comprehensively treat TPFs and reducing potential knee instability and secondary osteoarthritis (OA).

## Material and methods

A retrospective study was conducted on a consecutive series of 57 patients with a TPF admitted at our Orthopedics and Trauma Department between January 1st, 2022, and December 31st, 2022.

### Inclusion and exclusion criteria

Inclusion criteria were unilateral TPFs, MRI, and CT of the knee performed before surgery, evidence of skeletal maturity, and patients with a joint incongruence of 2 mm in CT evaluation treated surgically. Exclusion criteria were patients who had not performed an MRI prior to surgery, open fractures, extraarticular metaphyseal fractures of the tibia, patients with previous ligament reconstruction, previous corrective osteotomies, previous traumatic surgical treatment around the knee, and previous systemic or local knee infections.

### Data extraction

For each patient included, the following data were analyzed and reported on a standard template: age; sex; side of the affected knee; AO/OTA [13, 14] and Schatzker [10] classification; overall, age-related, and sex-related incidence of STIs. The dataset was collected by two authors (AR and FG), and a third author (FB) was consulted to resolve any doubts.

### Imaging detection at admission

At the patient's admission to the emergency department presenting with a suspected TPF, two X-ray views were obtained: AP and lateral. If a fracture that satisfied the surgical criteria, including fracture displacement and articular incongruity of 2 mm, was confirmed, a knee CT scan was indicated. The CT examination was postponed only in those cases where there was the need to perform damage control orthopedic (DCO) surgery first. In these cases, external fixation to stabilize the knee was performed, and a further CT study was carried out. After X-ray and CT evaluation, two authors (AR and FB) assigned the most appropriate AO/OTA [13, 14] and Schatzker [10] classification type to the fracture pattern. A third author (SR) was consulted in cases of uncertainty.

Afterward, an MRI scan was performed on each patient before surgery as a standard of practice in our department. The MRI protocol (1.5 T, Siemens) included axial, coronal, and sagittal scans with standard and fast spin-echo (FSE) fat-suppressed T1- and T2-weighted sequences. These were analyzed by a radiologist experienced in musculoskeletal tissue injuries. Any associated STIs were examined, which included: anterior cruciate ligament (ACL), posterior cruciate ligament (PCL), medial collateral ligament (MCL), lateral collateral ligament (LCL), lateral meniscus (LM), and medial meniscus (MM). Among the ligamentous injuries, grade 1 was represented by a slightly abnormal signal but with evidence of most fibers intact. Grade 2 showed discontinuity of more than 50% of the fibers. Grade 3 represented the completely torn ligament and was evident by an extensive fiber injury. Bone avulsion injuries were defined as grade 3 injuries. Only grade 2 and 3 ligamentous injuries undergoing surgical treatment were included in the analysis. Meniscal injuries involve the anterior horn, body, posterior horn, anterior root, or posterior root of the meniscus. For meniscal tears, high signal intensity reaching the articular surface or contour abnormality was considered indicative of an injury, and those undergoing surgical treatment were included in the analysis.

All TPFs analyzed using the Schatzker [10] and AO/OTA [13, 14] classification were divided accordingly, and each ligamentous injury and associated meniscal damage was recorded. Finally, cases undergoing surgical treatment of the associated lesions identified without MRI support and cases undergoing surgical treatment of the associated lesions identified exclusively with MRI assistance, without which they would have remained unrecognized, were recorded.

### Ethical approval

This study was defined as exempt from IRB approval (retrospective study on a well-established surgical procedure) and was conducted in accordance with the ethical standards laid down in the 1964 Helsinki Declaration and its later amendments.

### Statistical analysis

The statistical analysis was conducted using SAS software (version 9.4, North Carolina State University, USA). A descriptive analysis was performed for demographic data, fracture classification type, and STIs reported. Categorical variables were measured using absolute frequencies and percentages. Continuous variables were calculated as median value and interquartile range (IQR). Quantitative variables were examined using the Mann–Whitney U test, and qualitative variables were analyzed using the chi-square test and Fisher's exact test based on the exact binomial distribution. A *p*-value < 0.05 was considered statistically significant.

## Results

This study included 57 TPF patients, with 24 right and 33 left operated knees. The male/female (M/F) ratio was 32:25. All patients were grouped according to AO/OTA [13, 14] classification into 41 type B (group 1, N:4,7%; group 2, N:12, 21%; group 3, N:8, 14%), and 41 type C (group 1, N:7, 12%; group 2, N:2, 4%; group 3, N:24, 42%), and according to Schatzker's [10] classification into type I (N:0, 0%), type II (N:2, 4%), type III (N:16, 28%), type IV (N:13, 23%), type V (N:7, 12%) and type VI (N:19, 33%). The demographic characteristics are shown in Table 1.

**Table 1:** Main demographic characteristics of patients included

Parameters		*N* (%)
Total N		57
Age, median (IQR)		36 (23–54)
Sex	F	25 (44%)
	M	32 (56%)
Side	L	33 (58%)
	R	24 (42%)
AO/OTA classification	41B1	4 (7%)
	41B2	12 (21%)
	41B3	8 (14%)
	41C1	7 (12%)
	41C2	2 (4%)
	41C3	24 (42%)
Shatzker classification	I	0 (0%)
	II	2(4%)
	III	16 (28%)
	IV	13 (23%)
	V	7 (12%)
	VI	19 (33%)

*N* number of cases, *%* percentage, *F* female, *M* male, *L* left, *R* right

Twenty-four patients underwent DCO on emergency department admission using an external fixator (Hoffmann® 3 MRI system, Stryker). The mean time to perform MRI after emergency department admission was 7.7 (min: 1; max: 24) days. The mean time to perform definitive surgery after emergency department admission was 15.6 (min: 4; max: 32) days. Some timing delays in performing MRI and operative procedures were related to extended intensive care unit (ICU) observation and clinical stabilization of patients with severe conditions involving other non-orthopedic pathologies or multiple fractures, including spine, pelvic ring, and acetabulum fractures.

The prevalence of associated STIs in overall, age- and sex-related settings are shown in Table 2.

**Table 2:** Prevalence of soft tissue injuries

Overall prevalence of soft tissue injuries				
Soft tissue injury	N / Total *N* (%)			
LM	26/57 (45.6%)			
MM	10/57 (17.5%)			
ACL	14/57 (24.6%)			
PCL	10/57 (17.5%)			
LCL	5/57 (8.8%)			
MCL	13/57 (22.8%)			
Prevalence of age-related soft tissue injuries				
Soft tissue injury	< 25 y.o., *N* (%)	25–45 y.o., *N* (%)	> 45 y.o., *N* (%)	*p*-value
LM	9 (35%)	9 (35%)	8 (30%)	0.63
MM	2 (20%)	4 (40%)	4 (40%)	0.83
ACL	4 (29%)	7 (50%)	3 (21%)	0.44
PCL	4 (40%)	2 (20%)	4 (40%)	0.46
LCL	1 (20%)	3 (60%)	1 (20%)	0.61
MCL	3 (23%)	6 (46%)	4 (31%)	0.79
Overall	16	21	20	
Prevalence of sex-related soft tissue injuries				
Soft tissue injury	F, N (%)	M, N (%)	*p*-value	
LM	9 (35%)	17 (65%)	0.29	
MM	5 (50%)	5 (50%)	0.74	
ACL	6 (43%)	8 (57%)	1	
PCL	5 (50%)	5 (50%)	0.73	
LCL	3 (60%)	2 (40%)	0.64	
MCL	3 (23%)	10 (77%)	0.12	
Overall	25	32		

*N* number of cases, *%* percentage, *LM* lateral meniscus tear, *MM* medial meniscus tear, *ACL* anterior cruciate ligament, *PCL* posterior cruciate ligament, *LCL* lateral collateral ligament, *MCL* medial collateral ligament, *y.o.* years old

Table 3 shows the prevalence of associated STIs according to the AO/OTA [13, 14] and Schatzker [10] classification.

**Table 3:** Prevalence of soft tissue injuries according to AO/OTA and Schatzker classifications

Soft tissue injury	AO/OTA classification		Schatzker classification						
	41B	41C		I	II	III	IV	V	VI
	N / Total N (%)	N / Total N (%)		N / Total N (%)	N / Total N (%)	N / Total N (%)	N / Total N (%)	N / Total N (%)	N / Total N (%)
LM	15/24 (63%)	11/33 (33%)		/	2/2 (100%)	9/16 (56%)	8/13 (62%)	1/7 (14%)	6/19 (32%)
MM	5/24 (21%)	5/33 (15%)		/	1/2 (50%)	2/16 (13%)	2/13 (15%)	1/7 (14%)	4/19 (21%)
ACL	6/24 (25%)	8/33 (24%)		/	0/2 (0%)	5/16 (31%)	5/13 (38%)	0/7 (0%)	4/19 (21%)
PCL	5/24 (21%)	5/33 (15%)		/	1/2 (50%)	3/16 (19%)	2/13 (15%)	0/7 (0%)	4/19 (21%)
LCL	0/24 (0%)	5/33 (15%)		/	0/2 (0%)	0/16 (0%)	0/13 (0%)	0/7 (0%)	5/19 (26%)
MCL	9/24 (38%)	4/33 (12%)		/	1/2 (50%)	7/16 (44%)	1/13 (8%)	1/7 (14%)	3/19 (16%)

*N* number of cases, *%* percentage, *LM* lateral meniscus tear, *MM* medial meniscus tear, *ACL* anterior cruciate ligament, *PCL* posterior cruciate ligament, *LCL* lateral collateral ligament, *MCL* medial collateral ligament

An important finding concerns surgical management after MRI is performed. The MRI routinary application allowed the identification of all STIs associated with TPF that may or may not have required surgical treatment. The relevant result from this observational study was that the number of additional surgical procedures performed following STIs was higher than the number of STIs that did not require surgical treatment. Specifically, statistical significance was found regarding the additional MRI detection of the surgically treated LM, ACL, PCL, and MCL injuries (*p* < 0.05). In contrast, the amount of additional MM and LCL injuries identified using MRI, which led to other surgical procedures, did not appear to be statistically significant (*p* > 0.05) (Table 4).

**Table 4:** Soft tissue injuries that required additional surgical procedures after detection using MRI

Soft Tissue Injury	No additional surgical procedure was performed *	An additional surgical procedure was performed **	Total number of soft tissue surgical procedures performed	OR	χ^2^	*p*-value
	N (%)	N (%)	N (%)			
Total N	31	26	57			
LM	8 (26%)	18 (69%)	26	5.14	12.11	**0.005**
MM	5 (16%)	5 (19%)	10	2.21	1.23	0.268
ACL	2 (6%)	12 (46%)	14	8.95	8.80	**0.003**
PCL	2 (6%)	8 (31%)	10	5.85	4.56	**0.033**
LCL	1 (3%)	4 (15%)	5	5.38	1.58	0.208
MCL	2 (6%)	11 (42%)	13	8.13	7.68	**0.006**

*MRI support was not necessary for the decision-making process; **MRI support was crucial to the decision-making process; *N* number of cases, *%* percentage, *LM* lateral meniscus tear, *MM* medial meniscus tear, *ACL* anterior cruciate ligament, *PCL* posterior cruciate ligament, *LCL* lateral collateral ligament, *MCL* medial collateral ligament, *OR* odds ratio, *χ*^*2*^ chi-square

## Discussion

**Fig. 1:**
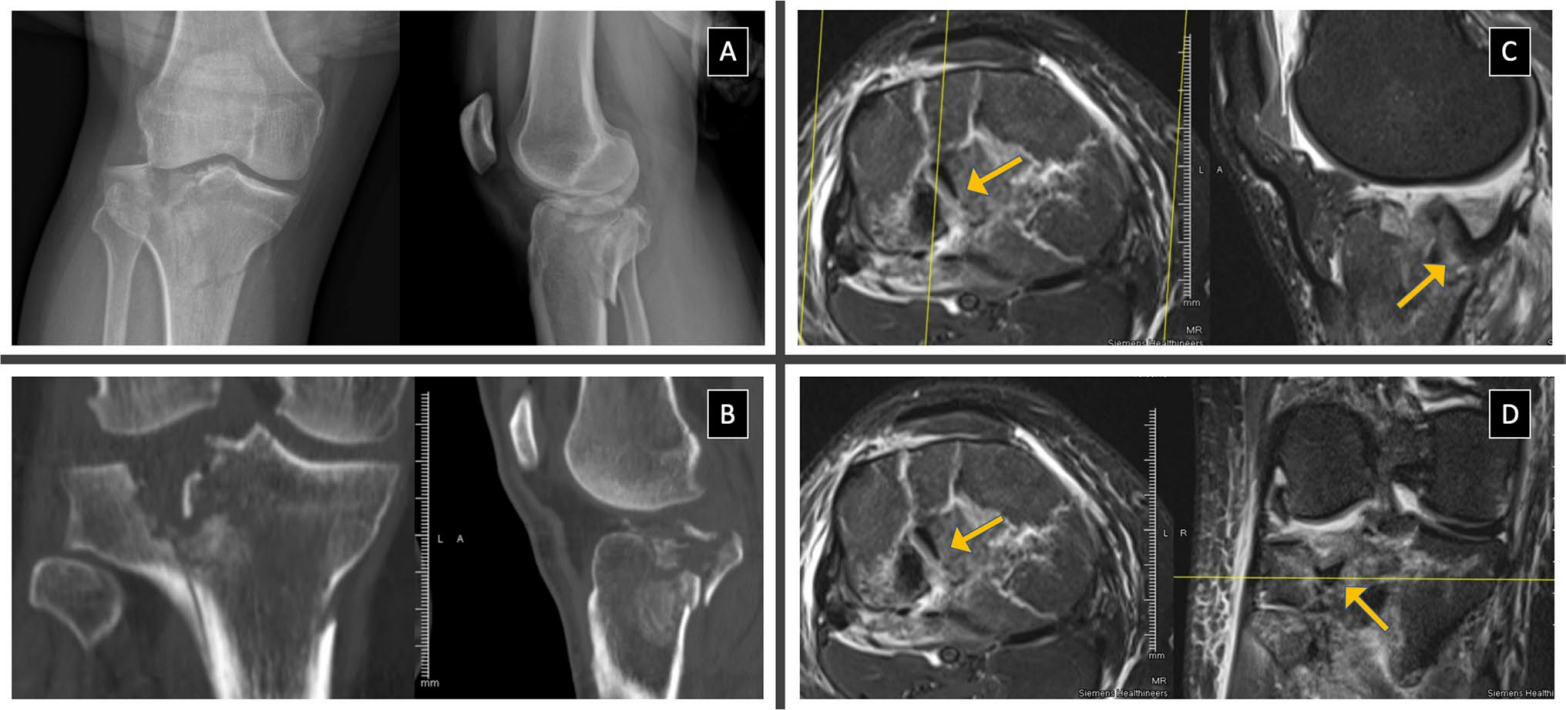
Multifragmentary tibial plateau fracture with postero-lateral depression and displaced lateral meniscus (LM) into the fracture. **A** anteroposterior (AP) and lateral X-ray views; **B**, Coronal and sagittal computed tomography (CT) scans; **C** Axial and sagittal T2-weighted fat-suppressed fast-spin-echo (FSE) sequences; **D** Axial and coronal T2-weighted fat-suppressed fast-spin-echo (FSE) sequences. The arrow (yellow) indicates the entrapment of the injured LM segment within the postero-lateral tibial plateau fracture

The most important finding is that MRI in TPFs led to a statistically significant number of additional procedures for associated STIs treatment that otherwise would not have been performed. The results reported in this study are consistent with those in the literature and demonstrate a high incidence of STIs associated with TPFs [3–6, 17, 18]. The MRI relevance in investigating TPFs-associated lesions was reported in several studies [3–6]. Yacoubian et al. [19], in their series of 52 TPFs, highlighted that MRI compared with X-ray and CT, increased both the interobserver agreement and the treatment plan among surgeons. MRI evaluation changed interobserver reliability and treatment approach to TPFs in a statistically significant 23% of cases. According to the authors, MRI revealed depression or comminution not detected by X-ray and CT and STIs that changed the operative approach. Therefore, the authors recommend MRI evaluation in all high-energy TPFs [19]. Sheperd et al. [20], in their series of 20 nondisplaced or minimally displaced TPFs analyzed with MRI, described the MRI importance in detecting associated STIs. According to the study, 98% of the nondisplaced TPFs had associated meniscal, cruciate, or collateral ligament tears; in 40% of cases, there was a complete ligament injury [20]. A similar study was conducted by Gardner et al. [2]. In their series of 103 TPFs, the authors examined the STIs incidence associated with TPFs. They described a complete ACL, PCL, LCL or MCL tear in 77% of cases. Specifically, 57% of patients reported either a peel-off or a complete ACL tear; a PCL injury was described in 28% of TPFs. An entire LCL lesion was shown in 29% of cases, while a complete MCL tear in 32% of patients. Finally, an LM tear was reported in 74% of TPFs, while a MM one in 44% of cases [2]. Associated STIs management in TPFs is still debated. The treatment rationale is to restore the knee stability and its natural biomechanics to avoid complications like postoperative knee instability, joint locking, swelling, and early posttraumatic OA that may reduce patients' outcomes [1, 7–9, 21]. While some authors did not report improved short-term outcomes in patients undergoing TPFs and associated soft tissue treatment [22], others described good clinical outcomes in patients treated for both TPFs and meniscal or ligament lesions [23, 24]. In our Orthopedics and Trauma Department, STIs are performed concomitantly with TPFs, as recommended in several studies, to provide a unique surgical treatment while beginning an early rehabilitation program [7, 24]. MRI is routinely carried out, while arthroscopy is based on the fracture pattern and associated STIs treatment. Although ligamentous and meniscal injuries may be diagnosed arthroscopically, MRI was preferred because it improves operative planning, avoids surgical risks, and identifies all the possible soft tissue lesions keeping the investigation within acceptable time and cost, as reported by Crawford et al. in their systematic review comparing MRI and arthroscopy in the knee injuries diagnosis [17, 25]. A total of 26 patients of the 57 included in the study underwent additional procedures for STIs treatment during TPFs. A statistically significant difference was demonstrated for LM lesions, with 18 patients experiencing mainly posterior horn and root injuries that required arthroscopic repair. Five patients with MM bucket-handle lesions underwent arthroscopic treatment. In some meniscal injuries, particularly of the LM, MRI revealed preoperatively the entrapment of the damaged meniscus in the TPFs. This lesion non-treatment may increase the risk of fracture healing failure because of the meniscus interposition in the fracture (Fig. 1). MRI also determined a statistically significant number of additional procedures for ACL- and PCL-associated injuries. Specifically, in 20 patients, MRI detected a partial injury or peel-off that was treated by repair or augmentation by internal bracing with a suture tape. Leading to a statistically significant number of additional procedures, the MRI identified 11 MCL lesions that underwent re-tensioning or reconstruction during TPFs treatment. Finally, four LCL lesions required reconstruction. Ligament treatment could increase knee stability, reducing further meniscal injury risk and secondary OA degeneration [7, 26].

This study has several strengths. It is the first, to date, to analyze the MRI impact in determining additional surgical procedures in TPF treatment. Second, the diagnostic protocol for TPFs performed in our Orthopedics and Trauma Department demonstrated how MRI is an acceptable, cost-effective technique that allows the identification of all associated STIs improving preoperative planning and focusing arthroscopy exclusively on lesions treatment. Third, the patient sample is large enough to be compared with other studies that have analyzed MRI in TPFs to investigate associated STIs.

This study also has several limitations. First, it is retrospective in design, and the nature of this study has intrinsic limits. Second, the sample considered in this study derived from a consecutive series of patients with a TPF admitted at our Orthopedics and Trauma Department and met the selected inclusion criteria; this could result in selection bias. Third, the study exclusively analyzed whether MRI implementation would result in an additional surgical procedure during the TPFs treatment. Pre- and postoperative clinical scores were not performed because they were not in this study's aim. Fourth, the number of TPFs included, although in line with other similar studies, may not be sufficient to reveal a correlation between the bone fracture pattern according to Schatzker [10] or AO/OTA [13, 14] classification and the associated STIs.

There is a strong correlation between STIs and TPFs. STIs increase in high-energy trauma; however, they are also observed in composed or minimally displaced TPFs; therefore, MRI would always be recommended in TPF cases [3–6]. It is unclear how untreated associated injuries can lead to knee instability, non-union, and OA progression. Although some authors emphasized that ligamentous and meniscal injury treatment do not significantly affect clinical outcomes, it should be underlined that their data is derived from a short-term analysis [22]. To date, no randomized clinical trials are comparing the medium- to long-term patients with TPFs who have undergone associated STIs treatment, so further studies with larger samples and longer follow-ups are needed to analyze the long-term effect of treating or not treating associated STIs in TPFs.

## Conclusions

TPFs are complex, comminuted intra-articular fractures that are typically associated with ligamentous and meniscal injuries. Preoperative MRI is an effective and affordable procedure that identifies all associated STIs. Its routine use has had a statistically significant impact on changing the surgical treatment of TPFs.

## Data Availability

Data is provided within the manuscript or supplementary information files.
